# RNA-Binding Macrocyclic Peptides

**DOI:** 10.3389/fmolb.2022.883060

**Published:** 2022-04-19

**Authors:** Sunit Pal, Peter ‘t Hart

**Affiliations:** Chemical Genomics Centre of the Max Planck Society, Max Planck Institute of Molecular Physiology, Dortmund, Germany

**Keywords:** macrocyclic peptides, RNA binding, structure-based design, peptide library screening, natural products

## Abstract

Being able to effectively target RNA with potent ligands will open up a large number of potential therapeutic options. The knowledge on how to achieve this is ever expanding but an important question that remains open is what chemical matter is suitable to achieve this goal. The high flexibility of an RNA as well as its more limited chemical diversity and featureless binding sites can be difficult to target selectively but can be addressed by well-designed cyclic peptides. In this review we will provide an overview of reported cyclic peptide ligands for therapeutically relevant RNA targets and discuss the methods used to discover them. We will also provide critical insights into the properties required for potent and selective interaction and suggestions on how to assess these parameters. The use of cyclic peptides to target RNA is still in its infancy but the lessons learned from past examples can be adopted for the development of novel potent and selective ligands.

## Introduction

**FIGURE 1:**
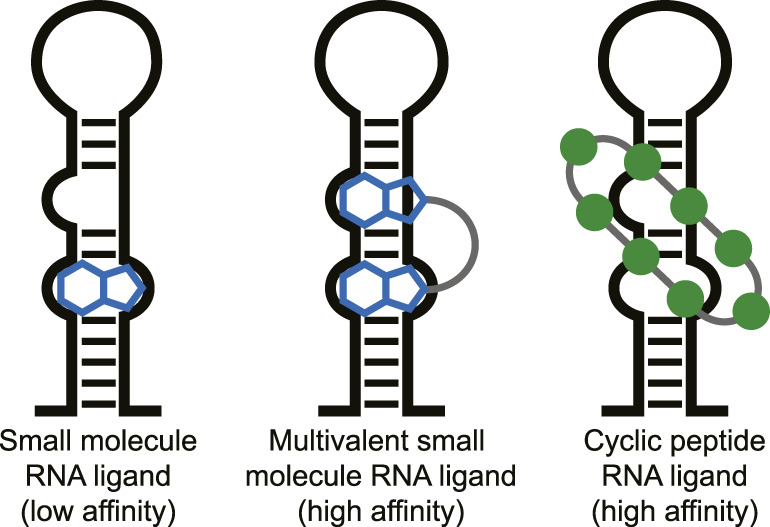
Different types of ligands can be used to target individual or multiple RNA secondary structures.

Targeting RNA for therapeutic effect has recently undergone a significant increase in attention. RNA in its various forms is involved in nearly all cellular processes potentially providing an abundance of therapeutic targets (Sharp, 2009; Donlic and Hargrove, 2018). Although RNA was long seen as an undruggable biomolecular target due to its intrinsic flexibility, various small molecules that potently bind to RNA have been described in the last years (Connelly et al., 2016; Disney et al., 2018; Donlic and Hargrove, 2018; Warner et al., 2018; Morgan et al., 2019). For effective small molecule binding it is important that the RNA has a stable tertiary fold and thereby produces pockets similar to classical protein pockets (i.e., riboswitches) (Blount and Breaker, 2006; Warner et al., 2018). However, targeting RNA without such stable tertiary folds with low molecular weight molecules (<500 Da) is still challenging. Selective and potent binding can be achieved with multivalent small molecules connected *via* a linker that target multiple secondary RNA structures simultaneously (Figure 1) (Gareiss et al., 2008; Luu et al., 2016; Velagapudi et al., 2016; Disney et al., 2018; Costales et al., 2020; Guan and Disney, 2012). The fact that larger multivalent molecules are required for effective targeting of RNA secondary structures suggests that cyclic peptides are also a suitable modality for this purpose. The extended interaction surface of a cyclic peptides also allows it to bind with multiple secondary RNA structure and achieve high potency and selectivity similar to the multivalent ligands (Figure 1). Indeed, various examples of RNA-binding cyclic peptide have been reported in the last 2 decades and will be reviewed here. We will discuss their structural elements, the strategy used to identify them, and their biological effects to provide a general view of successful strategies and applications. We categorized the reports into three categories that are defined by the method used to identify them: mimicking of RNA-binding proteins, peptide library screening, or as natural products.

### RNA as Target for Peptides

**FIGURE 2:**
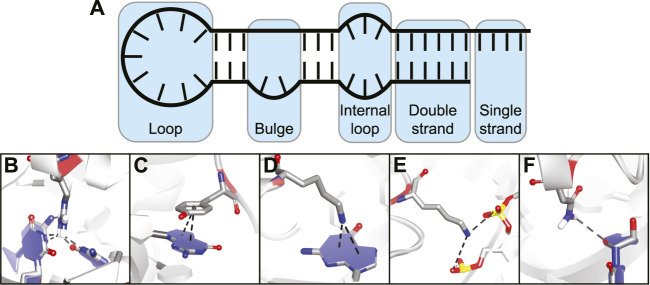
**(A)** Different secondary structural elements of RNA. **(B)** Hydrogen bonding interaction between amino acid and nucleotide base. **(C)** Stacking interaction between amino acid and nucleotide base. **(D)** Cation-π interaction between charged amino acid and nucleotide base. **(E)** Electrostatic interaction between charged amino acid and backbone phosphates. **(F)** Hydrogen bonding interaction between amino acid and nucleotide ribose.

The flexibility of an RNA molecule can make it difficult to target and depends on various factors such as its length and the occurrence of stable secondary structure elements including double strands, loops, internal loops, and bulges (Figure 2A) (Bevilacqua et al., 2016). On top of these secondary structure elements there are more complex tertiary structure elements within the RNA which include helical duplexes, triple strands, and pseudoknots (Butcher and Pyle, 2011). Tertiary structure elements can be quite stable intracellularly but stability is often lost *in vitro* due to the absence of molecular crowding and RNA-binding proteins (Spitale et al., 2015; Lu and Chang, 2016). Secondary structural elements on the other hand tend to be more stable *in vitro* and can be folded in a laboratory setting making screening and binding studies much more feasible. The easier tractability of secondary RNA structure as a target is also reflected in the fact that most targets for non-natural product RNA-binding peptides are short stem-loop structures such as those found in viral RNA and micro RNAs (miRNA) (Liu et al., 2016). However, a few examples exist of cyclic peptides that target more complex tertiary folds of RNA but these mainly include the antibiotic peptides binding to ribosomal RNA (rRNA).

The various interactions made between RNA-binding proteins (RBPs) and RNA provide both binding affinity and selectivity (Corley et al., 2020). Since peptides use the same basic building blocks, they can make similar interactions and essentially mimic RBPs. The types of interactions commonly observed between amino acids and nucleic acids can be divided into five categories:

- Hydrogen bonding interactions between polar amino acids or the peptide backbone and nucleotide bases (Figure 2B).
- Stacking interactions between aromatic amino acids and nucleotide bases (Figure 2C).
- Cation-π interactions between positively charged amino acids and nucleotide bases (Figure 2D).
- Electrostatic interactions between charged amino acids and the backbone phosphodiester (Figure 2E).
- Hydrogen bonding interactions between amino acid side-chains or peptide backbone and the nucleotide ribose (Figure 2F).

The first three categories provide binding affinity and nucleotide selectivity due to interactions that are specific for a nucleotide base. The last two categories provide binding affinity by interacting with parts that are the same for all of the nucleotides.(Gupta and Gribskov, 2011) Furthermore, they can also provide selectivity for a specific RNA structure by specific spatial orientation of the interactions. Interactions with the 2′-OH group can provide selectivity between RNA and DNA (Allers and Shamoo, 2001). By combining various interaction types in one peptide and positioning them correctly *via* cyclization, potent RNA-binding cyclic peptides can be obtained.

### Cyclic Peptides as RNA Binding Agents

Peptides are effective at interacting with shallow surfaces and grooves that are commonly found on RNA targets where pockets tend to be absent (Warner et al., 2018; Umuhire Juru and Hargrove, 2021). The ability to potently interact with featureless surfaces has been demonstrated by the numerous successes of peptides as potent inhibitors of protein-protein interactions (Pelay-Gimeno et al., 2015; Dougherty et al., 2017; Sawyer et al., 2017; Vinogradov et al., 2019; Wang X. et al., 2021). Cyclization of a peptide is often required for potent binding as is illustrated by the fact that the linear equivalents of these peptides typically bind with lower affinity due to their high flexibility and the therefore higher entropic penalty upon binding (Pelay-Gimeno et al., 2015; Wang H. et al., 2021; Bechtler and Lamers, 2021). A challenge in the development of a potent cyclic peptide is the need to identify the right cyclization strategy as incorrect conformational restraints can lead to a peptide that is not able to bind its target efficiently (Pelay-Gimeno et al., 2015). The variables that need to be considered are the connection points for cyclization and the chemistry used to do so and a variety of strategies has been applied to develop RNA binding cyclic peptides as will be illustrated in this review.

Besides higher affinity, cyclic peptides provide several other advantages that make them more suitable as ligands for intracellular targets. The elimination of *N*- and/or *C*-termini through cyclization makes the peptide less susceptible to recognition by exopeptidases (Chow et al., 2019; Vinogradov et al., 2019). Other cyclization strategies that do not eliminate the termini (side-chain to side-chain) are typically also effective at reducing proteolysis by constraining a cyclic peptide in a conformation that is not recognized by endopeptidases (Yudin, 2015). If peptides are to be used as drugs they will encounter such proteases both in the blood and in the cytoplasm making stabilization a necessity to achieve sufficient half-lives. To reach intracellular RNA targets it is also critical for RNA-targeting cyclic peptides to have sufficient membrane permeability. Although the rules to improve permeability are less well understood than proteolysis protection, there are various examples of membrane permeable cyclic peptides (Qian et al., 2017; Philippe et al., 2021). Cyclization can provide a way to force a peptide to form intramolecular hydrogen bonds and take up a conformation where hydrophobic side-chains shield the peptides polar groups in the hydrophobic membrane environment (Over et al., 2016; Rossi Sebastiano et al., 2018). The effective reduction in polar surface area allows the peptide to pass the membrane and a reversal of this effect occurs in a polar aqueous environment. However, this membrane passage method is only possible for peptides that almost exclusively carry hydrophobic side-chains which are not common for RNA-targeting ligands. Instead, membrane permeability for this class typically occurs *via* an endocytosis pathway and is driven by the presence of a high number of positive charges (mainly arginine) (Bechara and Sagan, 2013; Dougherty et al., 2019). Cyclization can also provide an increase in cellular uptake *via* this mechanism by optimally positioning these positive charges for membrane interaction (Lättig-Tünnemann et al., 2011; Qian et al., 2013). Various examples of cyclic peptides that target RNA that are likely using this mechanism of cellular uptake will be discussed below.

A few examples of cyclic peptides engaging intracellular targets exist that are either approved for clinical use or in clinical trials (Zhang and Chen, 2022). Romidepsin is a cyclic peptide natural product that targets histone deacetylases and its relatively small size (MW: 541 Da) allows it to pass membranes efficiently (Li et al., 1996; Nakajima et al., 1998). More striking is the membrane permeability and oral bioavailability of the much larger immunosuppressant cyclic peptides cyclosporin A and voclosporin. These 11 amino acid peptides use the intramolecular hydrogen bonding method described above to passively pass membranes and reach their intracellular targets (cyclophilin and calcineurin) (El Tayar et al., 1993; Geiger et al., 2022). Another interesting example is the hydrocarbon stapled peptide ALRN-6924 which is currently being tested in clinical trials and targets both MDMX and MDM2 which facilitate the degradation of the tumor suppressor protein p53 (Saleh et al., 2021). However, various other peptides have been shown to be orally bioavailable in animal studies indicating they possess a certain level of membrane permeability and could reach intracellular targets (Nielsen et al., 2017).

### Methods to Identify RNA-Binding Cyclic Peptides

The first hurdle to overcome when one wants to address an RNA target using cyclic peptides is to identify a suitable starting point. Various methods have been described to identify such starting points which we have divided in three categories:

1) RNA-binding cyclic peptides identified *via* mimicking of RNA-binding proteins.
2) RNA-binding cyclic peptides identified *via* library screening.
3) RNA-binding cyclic peptides identified as natural products.

For each method we will first discuss its specific advantages and disadvantages followed by examples from the literature which have been further categorized by the target RNA. Interestingly, various RNA targets have been explored by more than one approach allowing for a comparison to be made.

## RNA-Binding Cyclic Peptides Identified *via* Mimicking of RNA-Binding Proteins

**FIGURE 3:**
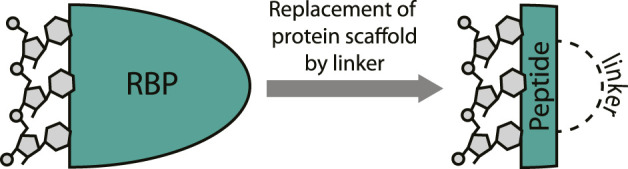
Excising a peptide from an RNA-binding protein provides a mimic that can target the RNA. A covalent linker replaces the protein fold.

It is not surprising that cyclic peptides can potently interact with RNA since they can effectively mimic the RNA-binding domain (RBD) of an RBP (Greenbaum, 1996). Various cyclic peptide examples discussed below will illustrate this concept as they are “excised” from RNA-binding proteins and maintain their RNA-binding capacity (Figure 3). The excision strategy is promising since it has been very successful for the inhibition of protein-protein interactions by mimicking one of the protein binding domains (Pelay-Gimeno et al., 2015; Sawyer et al., 2017). Lessons and strategies from this field can be extrapolated to the targeting of RNA and have proven fruitful in various cases. The structure-guided design process of RNA-binding cyclic peptides starts with an existing crystal or NMR structures of the target protein-RNA interaction. Both sequence and the fold of the peptide domain are important to achieve binding efficiency similar to native protein and choosing the correct cyclization strategy to achieve this is essential (Pelay-Gimeno et al., 2015). Once the correct macrocycle has been identified, further optimization can be achieved by point mutations.

### Transactivation Response RNA Binding Cyclic Peptides

**FIGURE 4:**
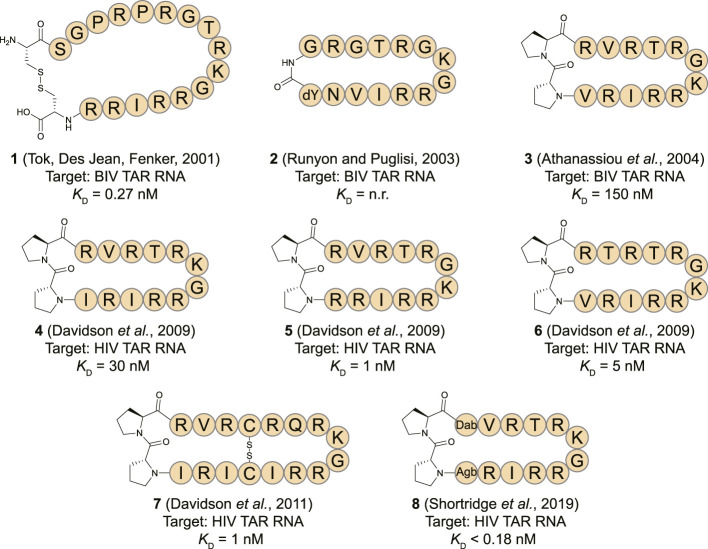
Structures of HIV TAR RNA binding cyclic peptides derived from the BIV or HIV Tat proteins. Dab = L-2,4-diaminobutyric acid, Agb = L-2-amino-4-guanidinobutyric acid (norarginine). n. r. = not reported.

The human immunodeficiency virus (HIV) transactivation response (TAR) element RNA has been widely explored as a target for macrocyclic peptides and is found in the 5′ untranslated region of the HIV mRNA (Ott et al., 2011). The TAR stem-loop is bound by the Tat protein which binds the positive transcription elongation factor P-TEFb to recruit the super elongation complex (SEC) and release RNA polymerase II for transcriptional elongation (Peterlin and Price, 2006; Gu et al., 2014). Due to a lack of structural information on the HIV Tat-TAR interaction the first reports of cyclic peptides targeting the TAR stem-loop focused on the analogous bovine immunodeficiency virus (BIV) Tat-TAR interaction (Pallansch et al., 1992; Tok et al., 2001). The BIV Tat domain binds the TAR sequence in a β-turn conformation forming a suitable starting point for cyclic peptide design (Puglisi et al., 1995). The Fenker group reported the disulfide bridged cyclic peptide **1** (Figure 4) by starting from the 17 amino acid WT sequence and incorporating two additional cysteine residues at the *N*- and *C*-terminus so that cyclization could be achieved by oxidative disulfide bond formation (Tok et al., 2001). The cyclic peptide was able to bind the TAR RNA with a *K*_D_ of 0.27 nM according to a fluorescence polarization (FP) assay. In comparison, the equivalent linear peptide had a *K*_D_ of 1.32 nM indicating cyclization led to a 5-fold improvement. A similar peptide (**2**, Figure 4) was later reported by Runyon et al. that was stabilized by grafting a β-turn sequence (D-Tyr-Gly) derived from the antibiotic peptide Gramicidin S onto the BIV Tat peptide and cyclizing it in a head-to-tail fashion (Runyon and Puglisi, 2003). The affinity of this peptide was not reported, but nuclear magnetic resonance (NMR) studies indicated it binds the BIV TAR RNA in a similar range as the linear peptide. Since circular dichroism (CD) spectroscopy studies indicated that the cyclic peptide adopted a random coil conformation in solution, it is unlikely that this cyclization strategy was effective to stabilize the β-turn.

**FIGURE 5:**
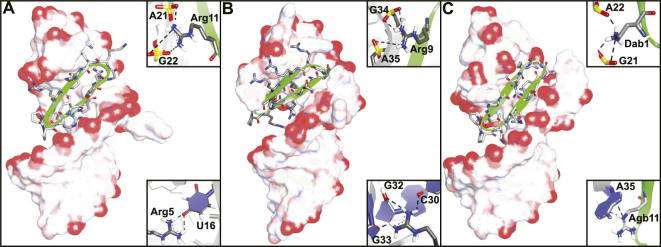
NMR structures of **(A)** peptide **3** bound to BIV TAR RNA (PDB ID—2A9X), **(B)** peptide **4** bound to HIV TAR RNA (PDB ID—2KDQ) and **(C)** peptide **8** bound to HIV TAR RNA (PDB ID—6D2U). Inlays depict specific interaction important for binding.

The Varani and Robinson groups developed TAR RNA binding cyclic peptides that improved in affinity with each iteration. The series of reports beautifully illustrates how structure based design can lead to highly potent ligands and started by introducing the efficient β-turn stabilizing motif D-Pro-L-Pro into the BIV Tat peptide (Athanassiou et al., 2004). They designed a small library by keeping the BIV Tat residues (72–79) the same but varying the remaining five positions in their peptide. Linear peptide synthesis was performed on solid support followed by mild acid cleavage to maintain side-chain protecting groups. These linear precursors were cyclized in a head-to-tail fashion in solution to yield the desired products. The best performing cyclic peptide (**3**, Figure 4) had a *K*_D_ of 150 nM for the BIV TAR RNA in an electrophoretic mobility shift assay (EMSA). The affinity was however 3-fold lower than a linear wild-type sequence but peptide **3** did display a strong selectivity for BIV TAR over HIV TAR (>10,000-fold difference). Surprisingly, NMR analysis showed that the β-hairpin peptide bound in the opposite orientation in comparison to the wild-type peptide it was derived from (Figure 5A) (Leeper et al., 2005). To convert **3** to a HIV TAR recognizing ligand they synthesized variants with various point mutations using the same head-to-tail cyclization strategy, leading to peptide **4**–**6** (Figure 4) (Davidson et al., 2009). Peptide **4** was found to have a binding affinity of 30 nM for HIV TAR as measured by EMSA while the similar **5** and **6** were even more potent with *K*_D_ values of 1 and 5 nM respectively. Interestingly peptide **3** and **6** only differ in position 2 and the valine to threonine mutation leads to a much more potent HIV TAR binding ligand. Although peptide **5** is even more potent, it does not display selectivity between HIV TAR and BIV TAR and binds both with equal affinity, potentially due to the increased charge. NMR studies showed that **4** bound both the apical loop as well as the bulge of the TAR RNA (Figure 5B), further illustrating that simultaneous binding to multiple secondary elements improves affinity and selectivity of the ligand. A single nucleotide mutation in the TAR RNA completely abolished binding highlighting the selectivity of the peptide. Further studies of **4** using fluorescence titration measurements showed that cyclization led to a more specific TAR RNA ligand in comparison to the linear Tat peptide (Lu et al., 2015). By generating a library of derivatives of peptide **4**, the optimized **7** was identified which had four amino acids inserted into the macrocycle including a central pair of cysteines that form an intramolecular disulfide bridge for further stabilization (Davidson et al., 2011). Peptide **7** has an affinity of 1 nM for HIV-TAR RNA reflecting a 30-fold improvement over the starting point **4** but similar to the previously identified **5**. Molecular dynamics simulations demonstrated that the Arg-Xxx-Arg-Lys motif found in both peptide **4** and **7** was essential for TAR RNA recognition (Li et al., 2013). The simulations indicated that the Lys residue is important for the correct positioning of the peptide, while both Arg residues form a sandwich that specifically recognizes G26 and G28 *via* hydrogen bond interactions. The peptides **4**, **5**, and **6** were further studied in cellular assays by first evaluating the cellular uptake of a fluorescently labelled variant of **6** (Lalonde et al., 2011). Since these peptides are derived from the Tat sequence which is well known to be able to pass the cell-membrane it was not surprising that efficient internalization was observed in hT4R5 fibroblasts. The inhibition of viral replication was measured for various HIV-1 isolates and found to be in the low micromolar range. Further evolution of peptide **4** was performed by preparing a new library of derivatives that incorporated non-proteinogenic amino acids at positions that seemed suitable for optimization based on the NMR structure (Shortridge et al., 2019). The introduction of L-2,4-diaminobutyric acid (Dab) at position 1 and L-2-amino-4-guanidinobutyric acid (Agb, norarginine) at position 12 led to peptide **8** which had an affinity <0.18 nM as determined by EMSA and 28 nM in a fluorescence emission assay using 2-aminopurine (2-AP) labelled TAR. Although peptide **8** has a very high affinity towards the TAR RNA, this did not translate to increased activity in cellular HIV replication and spread assays where it performed similar to peptide **4**. Although **8** is able to displace the Tat domain that binds TAR, it still allows other SEC components to bind the TAR apical loop allowing transcription elongation explaining the low cellular activity. The impressively high affinity can be explained by the high-resolution NMR analysis of **8** bound to the TAR RNA (Figure 5C) which demonstrated that the Dab is perfectly placed for an electrostatic interaction with two backbone phosphate groups while the Agb residue is placed ideally for a π-π interaction with adenosine 35. Peptide **8** also exemplifies that cyclic peptides can provide a potency that can’t be rivalled by monovalent small molecules as the most potent reported TAR ligand is the compound WM5 which has a *K*_D_ of 19 nM which is still two orders of magnitude away (Parolin et al., 2003).

**FIGURE 6:**
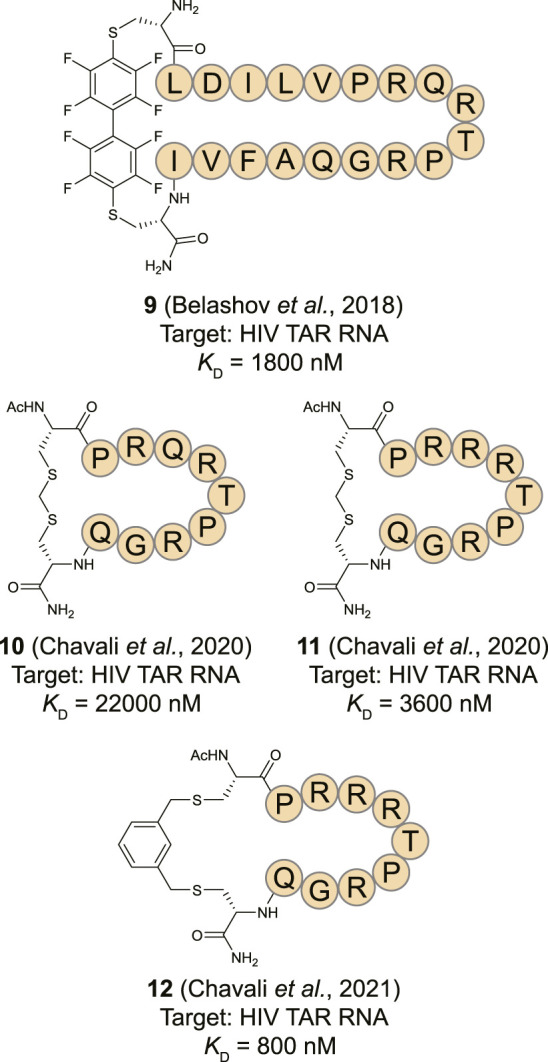
Structures of HIV TAR RNA binding cyclic peptides derived from RNA-recognition motif domains.

An alternative starting point for TAR RNA binding cyclic peptides was found in a lab evolved RNA recognition motif (RRM). The RRM is a common RNA-binding domain found in many RNA-binding proteins (Cléry et al., 2008; Daubner et al., 2013). To develop a TAR specific RRM, the spliceosomal protein U1A RRM was subjected to saturation mutagenesis and screened for binding to the TAR RNA stem loop allowing the identification of a new RRM with an affinity of 0.5 nM (Crawford et al., 2016). Structural studies of this new RRM in complex with the TAR RNA led to the observation that the main contribution to RNA binding was provided by a loop connecting two β-strands (Belashov et al., 2018). A cyclic peptide (**9**, Figure 6) was prepared from the β-strands and the loop by flanking the sequence with cysteines and cyclization using a perfluorobiphenyl linker. The peptide had an affinity of 1.8 µM towards 2-AP labelled TAR RNA and a shorter version was able to inhibit transcription of an HIV template using HeLa nuclear extracts and the Tat protein at a 20 µM concentration. Structural comparison of the loop from the evolved RRM to the previously described peptide **4** showed that the two have very different modes of binding. While both bind to the apical loop, peptide **4** also binds to the bulge potentially explaining its higher affinity and again illustrating the need for interaction with multiple secondary RNA structures for potent binding. Using an alternative cyclization strategy which connects the cysteine thiols *via* a methylene group, the same group prepared peptide **10** as a shortened cyclic peptide equivalent of **9** (Figure 6) (Chavali et al., 2020). At the same time they prepared peptide **11** by applying the same strategy but using a loop from a different RRM that was also able to potently recognize the TAR RNA. These peptides were cyclized by introducing two flanking cysteines and connecting them *via* a methylene bridge and had affinities of 22.0 and 3.6 µM, respectively. The more potent **11** was then optimized by exploring a variety of linkers to connect the cysteines leading to the meta-xylene cyclized peptide **12** with an improved binding affinity of 0.8 µM (Chavali et al., 2021). Peptide **12** was found to be inactive in a cellular assay for infectivity but alternative varieties were active albeit at relatively high concentrations >200 µM where they also started to show toxicity.

### RRE Targeting Peptides

Rev is a HIV protein that plays a regulatory role similar to the Tat-protein in the life cycle of the virus (Daly et al., 1989; Zapp and Green, 1989). The *N*-terminal arginine-rich motif (ARM) spanning residue 34–50 acts as both nuclear localization signal and RBD. The ARM is responsible for the regulatory activity of Rev by recognizing a cis-acting RNA target sequence known as the Rev response element (RRE) in the viral RNA and facilitating its transport to the cytoplasm for translation of viral proteins (Kubota et al., 1989; Cullen and Malim, 1991). The 17-residue long ARM domain exists in an α-helical conformation when bound to the RRE and has a high abundance of Arg residues to target the major groove of the stem part of the RRE (Kubota et al., 1989; Kjems et al., 1992; Tan et al., 1993). In contrast the Tat-Tar interaction, the REV-RRE interaction is multivalent where multiple REV proteins can bind the RRE RNA (Rausch and Grice, 2015).

**FIGURE 7:**
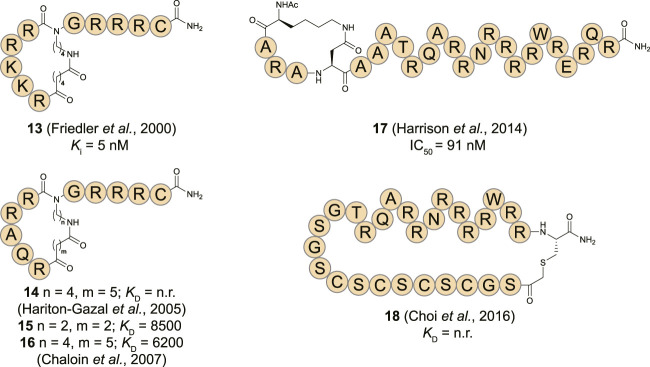
Structures of HIV RRE RNA binding cyclic peptides. n. r. = not reported.

By inhibiting the Rev:RRE interaction, viral replication can be reduced and the Loyter and Gilon groups reported the first cyclic peptides derived from the Tat ARM sequence to achieve this (Friedler et al., 2000). Interestingly, the Tat ARM efficiently inhibited the interaction between Rev and the RRE RNA which could be explained by the high homology between Tat and Rev which have similar regulatory activities in viral RNA maturation. Therefore, the Tat ARM was modified using a unique backbone cyclization strategy by connecting a diacid to the *N*-terminus, while changing a non-binding Gln in position 54 to a Gly and attachment of an amino linker to the backbone nitrogen. The amine linker and the *N*-terminal diacid were then connected for cyclization and a library was prepared by varying the lengths of these linkers. Peptide **13** (Figure 7) as well as other members of the library exhibited high affinity to the RRE RNA and inhibited the Rev:RRE interaction with low nanomolar (*K*_*i*_ = 5 nM) inhibition constants. Furthermore, a four-fold higher *K*_*i*_ value for the cyclic peptides in comparison to the linear Tat sequence (*K*_*i*_ = 20 nM) indicated that conformational restriction is indeed needed for better inhibitory activity. The same group later applied the backbone cyclization strategy to the Rev ARM as well to prepare a library of constrained peptides and investigated the effect on nuclear import and Rev-induced gene expression (Hariton-Gazal et al., 2005). Interestingly, all the cyclic peptides were able to inhibit the nuclear import ∼80–90% of Rev-GFP potentially due to competitive binding with importin β. Because of the high cell penetration levels of these peptides, they were further analyzed for their effect on Rev-induced gene expression. Among them, the peptide **14** modulated Rev-induced gene regulation most potently by reaching approximately 65% inhibition at a 4.6 μM concentration without displaying cellular toxicity. Later, the Devaux group extended this work to study *in vitro* inhibition of HIV-1 replication and to find the mode of inhibition of the cyclic peptides in the viral life cycle (Chaloin et al., 2007). Complete inhibition of HIV-1 replication in human H9 T-lymphocytic cells was found for two peptides (**15** and **16**) at a 50 μM concentration. Molecular modelling of RRE RNA—cyclic peptides complexes demonstrated that the peptides bind the major groove of the RRE RNA in a way that could interfere with the Rev-RRE interaction and thus reduce the level of transport of the full-length viral RNA in HIV-1 infected cells.

The interaction between the Rev ARM and RRE RNA follows an induced fit model as the isolated ARM domain rarely takes up an α-helical conformation in an aqueous medium (Bai et al., 2014). To achieve conformational preorganization, Harrison *et al.* applied a template-nucleated α-helix stabilization approach by introducing a constrained cyclic pentapeptide at the *N*-terminus of the isolated ARM domain (Maison et al., 2001; Patgiri et al., 2008; Harrison et al., 2014; Hoang et al., 2016; Pal et al., 2021). The cyclic pentapeptide itself was formed by a side-chain to side-chain lactam bridge between the Lys and Asp residues and exists as a stable α-helical turn in water which nucleates helix formation of a connected sequence. To avoid any undesirable steric clashes between the cyclic pentapeptide and the RNA surface, a computational analysis was performed to determine the optimal length of an alanine linker. Peptide **17**, with a triple alanine linker was found to maintain the correct distance while simultaneously maintaining helicity. The helix nucleator significantly improved the helicity from 6% for the linear peptide to 54% for peptide **17** as measured by CD spectroscopy. The binding affinity data corroborated with the CD data indicating an ∼10-fold enhancement of the IC_50_ values for peptide **17** compared to the linear peptide. Incorporation of the helix nucleating pentapeptide in the sequence of the helix itself rather than on the terminus led to highly stable helical peptides but required modification of essential residues reducing the affinity.

Choi *et al.* used the Rev ARM domain to prepare a cyclic peptide-gold nanoparticle hybrid material to target the RRE RNA *in vitro* and in live cells (Choi et al., 2016). The designed cyclic peptide **18** contains an α-helical region consisting of the Rev ARM domain and a cysteine-rich region for binding with the gold nanoparticles (AuNp). The α-helical and Cys-rich regions are separated by a flexible linker not to interfere with each other’s activity and folding behavior. The cyclic peptide was synthesized using a head-to-side-chain cyclization strategy by coupling bromoacetic acid to the *N*-terminus of the peptide followed by intramolecular *S*-alkylation of the free Cys thiol on solid support. CD spectrum analysis confirmed that the ARM domain adopts an α-helical conformation upon binding with AuNps. EMSA analysis showed comparable affinity for both cyclic and linear peptide/AuNps hybrid material with the stem-loop IIB region of monovalent RRE RNA. The cyclic peptide hybrid material showed better binding with multivalent RRE RNA than the linear counterpart, but exact affinities could not be determined. The interaction between the peptide hybrid material and multivalent RRE RNA was demonstrated to be quite specific as a mutant peptide hybrid material in which essential amino acids were replaced by alanine showed reduced binding and poor selectivity. Cellular assays demonstrated that the cyclic version was able to recognize RRE RNA both in the cytoplasm and the nucleus while the linear equivalent was only capable of nuclear detection.

### miRNA Targeting Peptides

**FIGURE 8:**
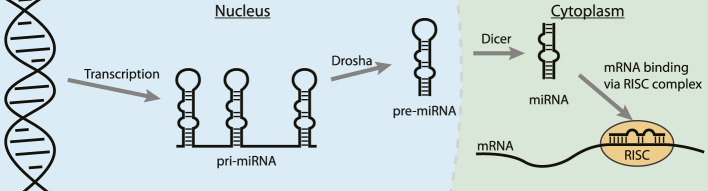
The steps in the biogenesis of miRNA. Binding to a pre-miRNA can block the pathway and inhibit miRNA downstream effects.

MicroRNAs (miRNA) are short non-coding RNAs (∼22 nt) that usually interact with the 3′ untranslated region (3′UTR) of mature mRNAs and induce degradation or translational repression (O’Brien et al., 2018; Gebert and MacRae, 2019). Abnormal expression of miRNAs can contribute to the progression of many cancers and they have therefore emerged as a novel class of therapeutic targets (Calin and Croce, 2006; Meng et al., 2007; Frankel et al., 2008; Ventura et al., 2008). After expression in the nucleus in the form of a primary miRNA (pri-miRNA) the nuclease Drosha trims the 5′- and 3′-ends to form the pre-miRNA (Figure 8) (Gebert and MacRae, 2019). Next, the pre-miRNA is transported to the cytosol where the RNAse Dicer cleaves the apical loop to form the mature miRNA. The mature miRNA can then be loaded on the RISC complex to bind its target mRNAs and either repress translation or promote degradation (van den Berg et al., 2008). One of the strategies to inhibit oncogenic miRNA activity is by interfering with the maturation process by binding to the precursor microRNA (pre-miRNA) hairpin to block both Drosha and Dicer processing (Li and Rana, 2014; Gebert and MacRae, 2019). The stable hairpin pre-miRNAs contain various RNA secondary structures that can be targeted by small molecules or peptides for potent binding and inhibition of maturation (Liu et al., 2016).

MiR-21 is a well-studied microRNA for its ubiquitous role in many types of cancer where its overexpression leads to down-regulation of tumor-suppressor and pro-apoptotic proteins (Jazbutyte and Thum, 2010; Pfeffer et al., 2015). Targeting pre-miR-21 is challenging as it contains a large unstructured loop which can be hard to address with small molecules making peptides an interesting alternative. Varani and co-workers reported that the β-hairpin cyclic peptide **5** which was originally developed to target TAR RNA as described above, also binds the stem-loop of pre-miR-21 with a *K*_D_ of 200 nM (Shortridge et al., 2017). Reduced affinity of **5** was observed for pre-miR-145 which is devoid of the helix region of pre-miR-21. Furthermore, pre-miR-18a was unable to replace pre-miR-21 from the peptide further illustrating a certain level of specificity for pre-miR-21. NMR analysis demonstrated large chemical shifts of protons of pre-miR-21 near the Dicer cleavage site upon titration with **5**. The NMR-derived structure of the peptide:RNA complex further revealed that the peptide interacts with the RNA at the major groove, however, the cyclic structure is flipped by 180° compared to the Tat ARM orientation on the surface of the TAR RNA. *In vitro* analysis demonstrated inhibition of Dicer processing by ∼ 50% at a concentration of 10 μM while *in vivo* analysis showed a concentration-dependent decrease of mature miR-21 in presence of **5** in HEK293 cells carrying an over-expressing miR-21 vector.

MiR-20b has also been described as an oncogenic miRNA by regulating the expression levels of hypoxia-inducible factor-1 alpha (HIF-1α) and vascular endothelial growth factor (VEGF) in various cancer types (Lei et al., 2009; Ahmad et al., 2015). To target miR-20b, the Varani group utilized a peptide isolated from the RRM domain of Rbfox2 which downregulates the maturation of pre-miR-20b by interacting with its apical loop (Sun et al., 2019). The essential amino acids (Asn151, Glu152, Arg153, and Gly154) of Rbfox2 RRM were retained in several cyclic β-hairpin peptide analogues that were constrained with the previously described D-Pro-L-Pro motif and prepared using the same synthetic strategy (Athanassiou et al., 2004). NMR analysis indicated that only one out of seven analogues had a stable structure in solution, but it was two others (**19** and **20**) that demonstrated the most potent affinity. Despite their higher degrees of flexibility, they were able to recognize the apical loop region of pre-miR-20b in an EMSA assay but accurate *K*_D_ values could not be determined and inhibition of either Drosha or Dicer processing wasn’t evaluated.

**FIGURE 9:**
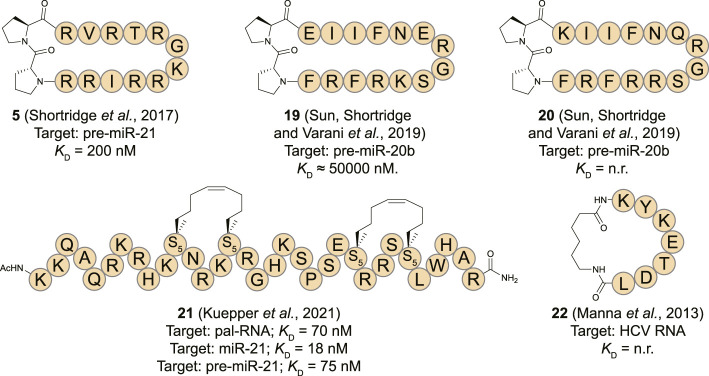
Structures of (pre-)miRNA and viral RNA binding cyclic peptides. n. r. = not reported. S_5_ = (*s*)-pentenylalanine.

Tomato aspermy virus 2b (TAV2b) protein is a suppressor of RNA silencing by binding the major groove of double stranded siRNA *via* its helix-loop-helix domain as was demonstrated by the crystal structure of the complex (Ding and Voinnet, 2007; Chen et al., 2008). Recently, Grossmann and his co-workers reported hydrocarbon stapled peptides derived from this domain to target the pre-miR-21 duplex (Kuepper et al., 2021). Hydrocarbon stapling is a highly effective strategy to stabilize α-helical conformations by introducing unnatural amino acids containing terminal alkenes into a peptide sequence and connecting these using ring-closing metathesis in a side-chain to side-chain fashion (Schafmeister et al., 2000; Cromm et al., 2015). CD spectroscopy analysis demonstrated an increase in helicity of a linear TAV2b peptide upon binding with the RNA indicating that hydrocarbon stapling could be a feasible strategy. A library of peptides with staples in different positions was prepared and EMSA and isothermal titration calorimetry (ITC) analysis showed that three peptides potently bound a palindromic RNA duplex used in the crystal structure that the peptides are derived from (Chen et al., 2008). The most potent of the stabilized peptides (**21**, Figure 9) featured a double staple and had a *K*_D_ of 0.07 μM which was significantly more potent than the linear equivalent (*K*_D_ = 1.19 μM) as determined by ITC. Since the TAV2b protein is known to non-specifically bind siRNAs, the authors hypothesized that the stapled peptide could also bind the highly analogous miRNAs. Interestingly, peptide **21** was able to bind miR-21 and pre-miR-21 with high affinity (18 and 75 nM, resp.) as measured by ITC and a biotinylated variant was able to pull-down these targets efficiently from K562 cells. Simulations demonstrated that peptide **21** bound to a site on pre-miR-21 that overlaps with the Dicer cleavage site which was confirmed by a biochemical pre-miR maturation assay. A series of RNA hairpins with different stems was used to test the selectivity of **21** which demonstrated that the peptide interacts with RNA in a non-specific manner similar to the parent TAV2b protein. Although the applicability of this peptide in its current form is limited due to its low selectivity, it is a feasible starting point for further optimization by incorporating modifications that can recognize specific RNA sequences.

Since the pri-miRNA processing Drosha binds the entire length of the stem, it is reasonable to expect that peptides **5** and **21** would inhibit this nuclease as well (Partin et al., 2020). Further cellular studies evaluating the accumulation of pri-miRNA and pre-miRNA would therefore be very informative but have not been reported yet.

### Other RNA Targets

The human La protein is associated with the viral life cycle of hepatitis C virus (HCV) and demonstrated to be a crucial structural element for internal ribosome entry site (IRES)-mediated translation of this virus (Pudi et al., 2003; Mondal et al., 2008). The RBD of La is involved in translational initiation of HCV viral RNA by promoting recruitment of the ribosome to the IRES without utilizing host cell initiation factors (Pudi et al., 2003; Hermann, 2016). To inhibit the interaction between the RBD of La and HCV IRES, Roy and his co-workers reported a cyclic peptide (**22**, Figure 9) derived from the RBD (Manna et al., 2013). The heptapeptide sequence (174-KYKETDL-180) adopts a β-turn conformation upon binding with the RNA which was cyclized by connecting the *N*- and *C*-terminus using an aminohexanoic acid linker. The peptide displayed concentration dependent translational inhibition of an HCV RNA luciferase reporter construct in Huh7 cells after 24 h. The effect was more pronounced for the cyclic peptide in comparison to the linear equivalent after extension of the treatment duration to 48 h which can possibly be explained by the higher resistance of cyclic peptides towards proteolysis. Viral protein inhibition followed the same pattern with the cyclic peptide being more potent than the linear construct. However, as no biophysical measurements are reported it is not possible to determine whether the improved activity of the cyclic construct is solely a result of increased stability or also an effect of increased binding affinity.

## RNA-Binding Cyclic Peptides Identified *via* Library Screening

**FIGURE 10:**
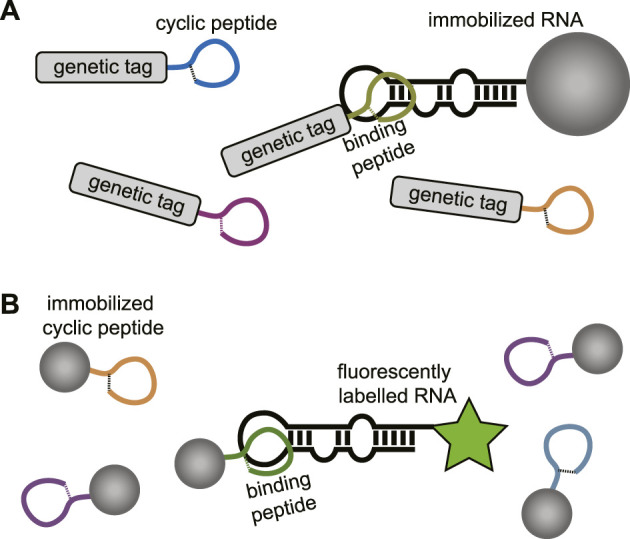
**(A)** Screening of genetically encoded peptides (i.e., mRNA or phage display). using an immobilized RNA target. **(B)** Screening of bead-bound synthetic peptides using a fluorescently labelled RNA.

In the absence of a structural model or to explore a wider chemical space for targeting a specific RNA cyclic peptide library screening can be applied. Modern cyclic peptide library screening technologies include mRNA display, phage display, SICLOPPS, and synthetic on-bead technologies which can provide access to large peptide diversity (10^5^–10^14^ members) (Passioura et al., 2014; Morioka et al., 2015; Liu et al., 2017). Besides the large library sizes, several of these techniques also allow the incorporation of unnatural amino acids which can be beneficial for improved binding affinity, selectivity, and stability of the peptide. The screening methods used to target RNA typically use an immobilized and a soluble component. For genetically encoded peptide libraries the RNA is immobilized, and the peptides are in solution (Figure 10A). The opposite situation is used for synthetic libraries where the peptides are immobilized, and the RNA is in solution (Figure 10B). The exploration of a wide chemical space also allows more insight in the peptide properties that are privileged for RNA binding. One can rapidly explore libraries with different macrocycle sizes but also side-chains carrying a variety of functional groups. The obtained information can be used to design new RNA focussed libraries and generate more potent and selective hits. Examples of cyclic peptide library screening against RNA targets are still rare but a few have been described and will be discussed below.

### Transactivation Response RNA Binding Cyclic Peptides

**FIGURE 11:**
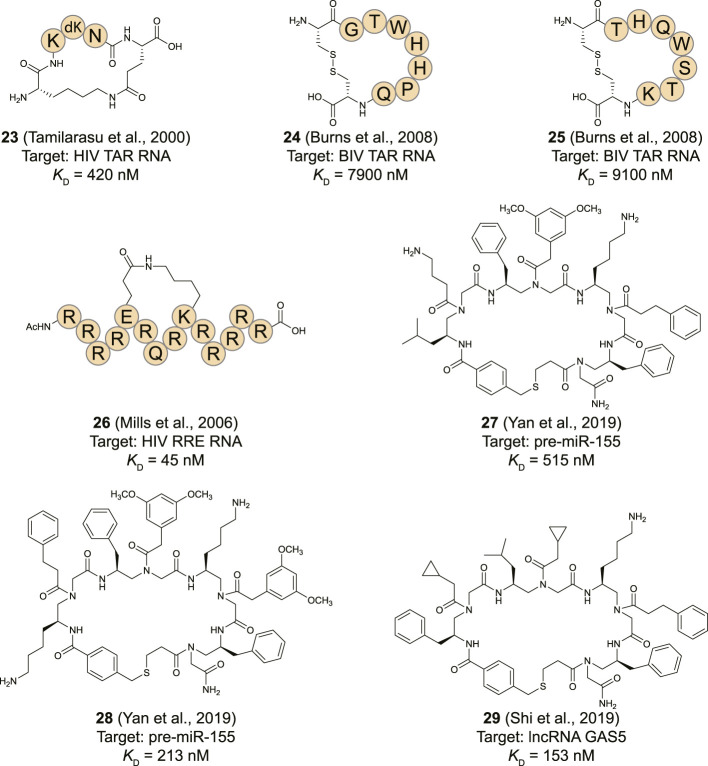
Structures of RNA-binding cyclic peptides identified via library screening.

Similar to the RNA-binding protein derived peptides described above, the TAR RNA has been used as a target for library screening. To target the TAR RNA, Hwang et al. used a combinatorial approach to generate a peptide library of tripeptide sequences (2.4 × 10^4^ members) followed by on-bead screening and identified a tripeptide (KdKN) that repressed Tat-induced trans activation in cells with an IC_50_ of approximately 50 nM (Hwang et al., 1999). Using these preliminary findings, Natarajan *et al.* sought to develop a cyclic peptide as an inhibitor of the HIV-1 Tat–TAR interaction by using the tripeptide as a starting point (Tamilarasu et al., 2000). They flanked the tripeptide with a lysine and a glutamic acid to form the cyclic KKkNE (**23**, Figure 11) pentapeptide by formation of a lactam bridge between the side-chains. The cyclic peptide demonstrated a 90% inhibition of Tat trans-activation at a concentration of 500 nM in a cellular reporter gene assay without affecting cell viability.

Due to the polycationic properties of peptides (**1**–**6**, Figure 4) derived from the BIV Tat ARM sequence they often face poor selectivity. The Melander group tried to overcome this issue by using phage display to screen a library of cyclic peptides (diversity 1.28 × 10^9^) against the BIV TAR RNA (Burns et al., 2008). The library was designed to contain peptides that have 7 randomized amino acids flanked by two Cys residues attached to the phage pIII coat protein. Cyclization *via* oxidative disulfide bond formation takes place spontaneously during phage assembly. After screening of the cyclic peptide library against biotinylated bTAR RNA, two cyclic peptides (**24** and **25**, Figure 11) were identified as hits with a *K*_D_ of 7.9 and 9.1 μM respectively for binding to bTAR using a fluorescence quenching assay. Both peptides were tested for binding to a randomized RNA sequence that is devoid of structure to probe their selectivity. Although peptide **24** could differentiate between the targets the affinity difference was only 2-fold while peptide **25** showed comparable binding to both targets. The hit peptides do not have a polycationic nature, indicating that potent binding can be achieved with alternative peptide properties. However, the lack of selectivity indicates further structural optimization is required to develop efficient RNA targeting cyclic peptides. The polycationic peptides benefit from an intrinsic membrane permeability which the peptides **24** and **25** are unlikely to have. Furthermore, disulfide bridges tend to be reduced in the cytoplasm causing such peptides to lose their cyclic structure after cellular uptake.

### Rev Response Element RNA Binding Peptides

The Frankel group used a genetic selection protocol to screen a library of poly-arginine peptides with mutations at a few positions to identify a peptide containing 13 arginine residues with a central glutamine that has high affinity for the RRE RNA (Tan and Frankel, 1998). The sequence was found to bind RRE in a helical conformation prompting optimization by a helix stabilization strategy (Mills et al., 2006). A library of peptides cyclized via side-chain to side-chain macrolactamization using a Lys/Glu pair or a Lys/Asp pair in various positions was produced and evaluated for binding to the RRE RNA by EMSA. One peptide (**26**, Figure 11) was found to bind RRE with a *K*_D_ of 45 nM which was 3-fold better than the linear equivalent and 2-fold better than the native Rev sequence. CD spectroscopy analysis indicated peptide **26** has a 33% helical conformation in solution which was surprisingly lower than the linear equivalent which demonstrated a 40.5% helicity (Tan and Frankel, 1998). Although the affinity of **26** for a truncated RRE element was 26 times lower, the high polycationic nature of the peptide is likely to lead to non-specific nucleotide binding.

### MiRNA Targeting Peptides

The stable stem-loop structures of miRNA make them attractive targets for library screening techniques as they can easily be maintained in the correct fold during selection. An affinity-based screening protocol to identify macrocyclic peptide inhibitors to target pre-miR-155 was reported by Yan et al. (2019) Downregulation of miR-155 was previously found to reduce breast cancer cell growth and therefore considered to be an attractive target (Mattiske et al., 2012). A library (3.2 × 10^5^) of macrocyclic peptides was prepared *via* split-and-pool synthesis using the one bead two components (OBTC) method containing various γ-substituted-*N*-acylated-*N*-aminoethylamino acids (γ-AA) as building blocks (Shi et al., 2017). To facilitate cyclization one of the backbone amines was modified with a protected thiopropionic acid group while the *N*-terminus was modified with bromomethyl benzoic acid. After removal of the thiol protecting group a straightforward nucleophilic substitution reaction under near neutral conditions yielded the cyclic peptides in close to quantitative yields. The variable side-chains used in the library included aliphatic amines, carboxylic acids, various cyclic and non-cyclic aliphatic chains and substituted and non-substituted aromatic rings. The on-bead library was exposed to fluorescently labelled pre-miR-155 followed by manual isolation of the most intense fluorescent beads and identification of the peptide. Although the cyclic peptide on the bead can’t easily be analyzed, the second component on the bead is a linear peptide that reports on the sequence of the cyclic peptide and can be analyzed *via* MALDI MS/MS. Two hits were identified from library screening and a FP assay indicated nanomolar binding affinities of peptides **27** and **28** for pre-miR-155 (515 and 213 nM, respectively). RT-qPCR analysis after cell treatment showed peptide **27** reduced the expression level of miR-155 by ∼70%, however, peptide **28** did not have any effect despite its high binding affinity. The lack of cellular activity for peptide **28** can potentially be explained by interaction with a pre-miR-155 site other than the ones involved in its maturation process since it demonstrated high cell-permeability. The hypothesis was supported by the fact that even peptide **27** was not able to completely inhibit pre-miR-155 cleavage in an *in vitro* Dicer assay at high concentrations (30 µM), further indicating that **27** inhibits miR-155 activity at a different stage (potentially during Drosha catalyzed conversion of the pri-miR). Interestingly, peptide **27** demonstrated a high selectivity for pre-miR-155 amongst a panel of six other miRNAs as well as a significant change in the mRNA and protein levels of the miR-155 target FOXO3A in MCF-7 breast cancer cells. Treatment of these cells with a 30 µM concentration of peptide **27** led to a 70% reduction of the viability together with an increase in the rate of apoptosis.

### Other RNA Targets

Long non-coding RNA (lncRNA) has received an increasing amount of attention for its role in various biological processes (Lavorgna et al., 2016; Uszczynska-Ratajczak et al., 2018; Statello et al., 2021). Although lncRNAs resemble mRNA in length and undergo similar processing steps such as splicing and polyadenylation they have different functions as they have no coding capacity. By interacting with other RNAs, but also DNA and proteins they can exert influence on gene regulation at various levels (Engreitz et al., 2016; Statello et al., 2021).

The lncRNA GAS5 influences the expression of the insulin receptor and is depleted in patients with type 2 diabetes (Carter et al., 2015). The degradation is regulated by regulator of nonsense transcripts 1 (UPF1) and the Patel group looked for compounds that can inhibit the interaction between GAS5 and UPF1 to increase GAS5 levels (Shi et al., 2019). The same OBTC screening strategy as described above for pre-miR-155 was used to screen a library of 2.1 × 10^5^ macrocyclic peptides against a GAS5 fragment isolated from the 3′ end. One potent peptide (**29**, Figure 11) was found to have a *K*_D_ of 153 nM for the lncRNA GAS5 as determined by FP and confirmed by EMSA. Interestingly, the peptide was shown to be cell permeable by fluorescence microscopy and significantly increased (4-fold) the levels of GAS5 already at a 20 nM concentration in adipocytes while not affecting other lncRNAs (NEAT1 and MALAT1). Although the inhibition of UPF1 binding wasn’t directly proven, peptide **29** does have the desired effect of upregulating insulin receptor A and B as well as glucose uptake in diabetic adipocytes after cotreatment with insulin.

Peptide **27**–**29** are especially interesting since they lack the polycationic nature that most other RNA targeting peptides have and incorporate novel fragments. Furthermore, the peptoid like nature of these compounds reduces the number of hydrogen-bond donors which can aid with cell-permeability (Vinogradov et al., 2019). The cellular activity of both peptide **27** and **29** is highly encouraging as well as their nanomolar potency indicating that synthetic libraries of this kind are an attractive way to identify novel RNA ligands.

## RNA-Binding Cyclic Peptides Identified as Natural Products

Besides mimicking of RBPs and library screening, nature provides another source of cyclic peptides that target RNA. Various natural product cyclic peptides have been described and most of these are antibiotic peptides that inhibit protein synthesis by binding to ribosomal RNA (rRNA) subunits (Poehlsgaard and Douthwaite, 2005). Although examples of other targets are still rare, natural products have served as scaffolds for modification towards RNA binding by applying medicinal chemistry approaches to alter the properties of a natural product scaffold.

### Ribosome Targeting Cyclic Peptides

Some of the most potent RNA binding cyclic peptides have been identified as inhibitors of the bacterial ribosome (Poehlsgaard and Douthwaite, 2005; Polikanov et al., 2018). The ribosome synthesizes proteins by simultaneously decoding the incoming mRNA and catalyzing amide bond formation by connecting amino acids loaded onto transfer RNA (tRNAs). The ribosome consists of ribosomal proteins but also for a large part of ribosomal RNA (rRNA) which forms the target for several cyclic peptide antibiotics (Nissen et al., 2020). Binding of antibiotic peptides to the ribosome blocks bacterial protein synthesis leading to reduced cell growth and potentially cell death. The availability of several high-resolution crystal structures of the bound peptides makes these antibiotics an interesting case to learn how peptides bind RNA. However, it has to be noted that the ribosome forms a unique RNA target due to its highly stable fold which is more comparable to a protein target than other types of RNA. Still the types of interactions involved can be informative in the design of RNA targeting peptides.

**FIGURE 12:**
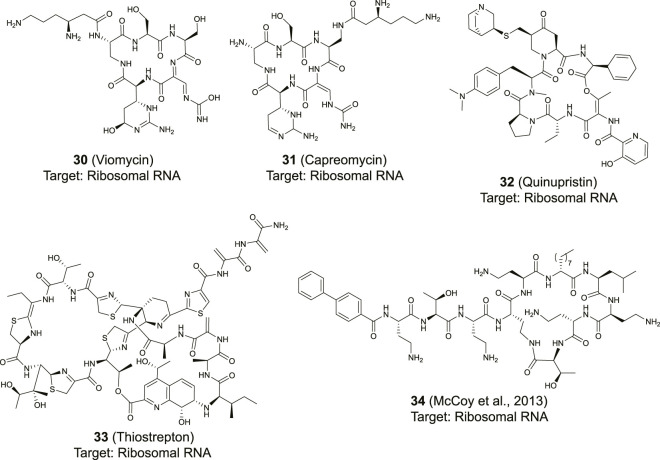
Structures of cyclic peptides natural products that target the ribosome.

**FIGURE 13:**
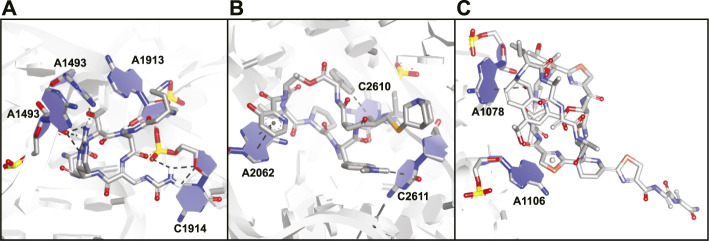
Crystal structures of antibiotic peptides bound to the ribosome. **(A)** Capreomycin (**31**) bound to the 16S rRNA (PDB ID—4V7M). **(B)** Quinupristin (**32**) bound to the 23S rRNA (PDB ID—4U26). **(C)** Thiostrepton (**33**) bound to the 23S rRNA (PDB ID—3CF5).

Viomycin (**30**) and capreomycin (**31**) are analogous cyclic peptides (Figure 12) produced by various *Streptomyces* species and used as drugs to treat tuberculosis (Thomas et al., 2003). They share the same binding site on the ribosome and contact both the small 30S subunit and the large 50S subunit (Stanley et al., 2010). The affinity of viomycin is greatly increased when a tRNA is bound to the ribosome and the interaction causes conformation stabilization of the entire ribosome stalling translation (Holm et al., 2016). Both viomycin and capreomycin contain a cyclic pentapeptide core including the amino acids serine, diaminopropionic acid, capreomycidine, and β-ureidodehydroalanine (Figure 12). The macrocycle is formed *via* a side-chain to tail type cyclization involving one of the diaminopropionic acids and the *C*-terminus. On the outside of the cyclic core there is a β-lysine in a different position for either viomycin or capreomycin. In the crystal structure of capreomycin bound to the ribosome (Figure 13A) it is visible that the unique amino acid capreomycidine forms a salt bridge with a backbone phosphate on the 16S RNA while simultaneously forming a hydrogen bond with a nearby adenosine *via* its guanidine group. The other non-proteinogenic amino acid β-ureidodehydroalanine also interacts with a backbone phosphate clearly demonstrating how these two specialized amino acids are essential for RNA binding.

Streptogramins are antibiotic peptides also produced by *Streptomyces* species that target the large 50S subunit of the ribosome and are typically produced as pairs (Poehlsgaard and Douthwaite, 2005; Polikanov et al., 2018). The pair consists of one polyketide/peptide hybrid macrocycle (streptogramin A) and one macrocyclic depsipeptide (streptogramin B) and the dalfopristin/quinupristin combination (also known as Synercid) has been approved for clinical use (Li and Seiple, 2017). Together, the compounds block the site where peptide bond formation occurs in the ribosome as well as the exit tunnel for the newly formed peptide chain (Harms et al., 2004; Noeske et al., 2014). Since dalfopristin has a very limited peptidic character we will only consider quinupristin (**32**, Figure 12). Similar to viomycin, quinupristin contains various non-proteinogenic amino acids that are important for interaction with the target RNA. The hexapeptide macrocycle is formed *via* a depsipeptide linkage connecting the *C*-terminus with the hydroxyl group of a dehydrothreonine residue. A high resolution crystal structure (Figure 13B) demonstrates that quinupristin interacts with the 23S rRNA mainly through stacking interactions of the aromatic side-chains (Noeske et al., 2014). These include stacking of the hydroxypicolinic acid that is connected to the backbone threonine and adenosine 2,062 as well as an edge-on stacking interaction between dimethylaminophenylalanine and cytosine 2,611. In contrast to viomycin most interactions are made with the nucleotide bases rather than the RNA backbone phosphates.

The thiopeptides comprise a large class of ribosome binding antibiotic peptides that are characterized by highly modified structures including several backbone heterocycles (Bagley et al., 2005). The most well studied member of this class is thiostrepton (**33**, Figure 12) which has a complex structure containing a central dehydropiperidine that connects two loops as well as a tail. The crystal structure of thiostrepton bound to the ribosome illustrates it binds to both the 23S rRNA and the ribosomal protein L11 (Figure 13C) (Harms et al., 2008). Binding of thiostrepton sterically blocks various translation factors from recruiting the ribosome and therefore initiation complex formation (Harms et al., 2008; Polikanov et al., 2018). Upon binding it forms a globular structure that interacts with several purine type nucleotides on the 23S RNA. The quinaldic acid heterocycle included in loop 2 of thiostrepton interacts with adenosine 1,078 by formation of a hydrogen bond while one of the backbone thiazoles stacks on top of adenosine 1,106. Although thiostrepton provides interesting RNA-binding properties, the high degree of modification makes it hard to synthesize and derivatize or to incorporate its features into other peptides.

Polymyxins are polycationic macrocyclic peptides that include multiple diaminobutyric acid residues in a seven amino acid loop, three exocyclic amino acids and a lipid tail (Poirel et al., 2017). The loop is formed *via* an amide connection between the side-chain amine of one of the diaminobuteryic acid residues and the *C*-terminus. Although polymyxins are generally thought to exert their antibiotic effect *via* a membrane disrupting or pore forming mechanism, they’ve recently been shown to interact with ribosomal 16S RNA (Mccoy et al., 2013; Poirel et al., 2017). The effect of a library of polymyxin derivatives was measured by displacement of the known 16S binding aminoglycoside kanamycin using a small 16S construct (Mccoy et al., 2013). Derivative **34** (Figure 12) was able to displace kanamycin with an IC_50_ in the low micromolar range but since other ribosomal components were missing from the measurement it is questionable whether this interaction is specific and part of the actual mode of action of the polymyxins. The interaction with rRNA is likely due to the high positive charge and therefore non-specific but the polymyxins might provide a starting point for preparing derivatives with more specific RNA binding properties.

### Other Targets

**FIGURE 14:**
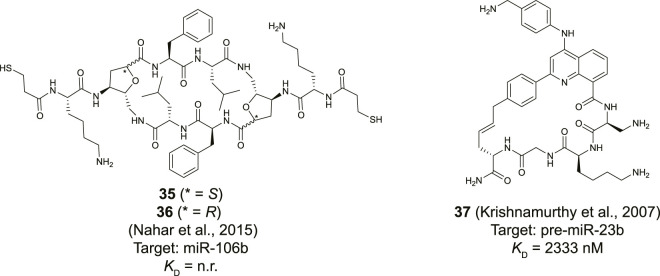
Structures of natural product derived cyclic peptides targeting RNA. n. r. = not reported.

Maiti and his co-workers reported a library of cyclic cationic peptides containing sugar amino acids to target miRNA (Nahar et al., 2015). The design of the peptide was inspired by the cyclic structure of the antibiotic decapeptide Gramicidin S. Several of the amino acids on both ends of the macrocycle were replaced with sugar amino acids (Saa) and further modified on both sides with an exocyclic lysine and a thiopropionic acid (**35** and **36**, Figure 14). The peptides were assembled using a previously described cyclodimerization of H_2_N-Saa(Boc)-Phe-Leu-OH followed by side chain deprotection and coupling of the exocyclic lysine and thiopropionic acid (Pal et al., 2011). Real-time qPCR-based profiling identified a significant change in the expression levels of miR-106b and miR-30b in MCF-7 cells after exposure to either **35** or **36**. The cyclic peptides were selective to pre-miR-106b of the pre-miR-106 family in an *in vitro* Dicer cleavage assay which is potentially due to the extra bulge in the stem region of pre-miR-106b but *in vitro* binding studies are lacking to further verify this hypothesis.

Although not directly identified as natural products, Beal and co-workers introduced a natural product inspired 2-phenylquinoline core into macrocyclic helix threading peptides (HTPs) to target RNA (Figure 14, compound **37**) (Krishnamurthy et al., 2007). The heterocyclic core intercalates with the RNA duplex of a stem-loop structure and the positively charged peptide further helps to stabilize the interaction with the RNA groove. The allyl modified heterocyclic core was coupled to the *N*-terminus of a peptide using an SPPS protocol, followed by cyclization with an allylglycine residue at the *C*-terminus using ring-closing metathesis conditions. Ribonuclease footprinting was used to determine RNA binding efficiency of the HTPs by measuring the protection against ribonuclease V1 cleavage of three different stem-loop RNAs that contained the intercalation target site. Peptide **37** was found to most potently interact when the intercalation site was flanked on the 3′-side with a large bulge (*K*_D_ = 52 nM). A smaller bulge was tolerated (*K*_D_ = 194 nM) but changing the bulge from the 3′ to the 5′ side abolished binding (*K*_D_ >10,000 nM). Interestingly, the linear counterpart of peptide **37** was 6-fold less potent confirming that cyclization improved binding. The stem-loop of pre-miR-23b contains the motif of the intercalation site flanked by a small bulge and peptide **37** was found to bind this RNA albeit with a more moderate affinity (*K*_D_ = 2.3 µM). Nonetheless, the method can be used to design peptides that selectively target certain RNA motifs by providing binding potency *via* the quinoline core and selectivity *via* the peptide.

## Discussion

The examples described above provide an exhaustive overview (with the exception of the antibiotic peptides) of known RNA-targeting macrocyclic peptides identified *via* three different methods. Each method has proven to be applicable to identify potent ligands and has its own advantages and disadvantages. Although the mimicking of RBPs has been the most widely explored strategy, it is limited to the available structural information. Generating conformationally constrained cyclic peptides with high affinity and selectivity requires a thorough understanding of the peptide fold upon binding with the RNA. Furthermore, the structure-based conversion of a linear peptide to a potent cyclic peptide followed by optimization *via* point mutations is a long and work intensive process as is illustrated by the TAR RNA binding peptides **3**–**8** whose reports span 15 years to achieve three orders of magnitude improvement in affinity. An interesting source for novel RBDs to excise peptides from could be provided by intrinsically disordered regions (IDRs). Various RBPs do not contain RNA-binding domains with stable folds but instead interact with their target RNAs through IDRs (Zeke et al., 2022). Recent advances in the structural characterization of these might provide novel starting points for cyclic peptide development.

The lack of structural information on therapeutically relevant protein-RNA interactions makes screening strategies an attractive alternative to identify cyclic peptide ligands for such RNAs. Although the examples are very limited, the advancement of screening techniques together with optimization of the intrinsic RNA-binding properties of the peptides in a library can provide potent and selective ligands. When designing a library one can also attempt to maintain properties that promote cellular uptake which is critically important to reach RNA targets. Peptides **27**–**29** clearly demonstrate that this is feasible, and that interesting biological activity can be achieved. Alternatively, peptides derived from proteins with promiscuous but potent RNA-binding properties such as the TAV2b derived cell permeable stapled peptide **21** could also provide an excellent starting for library creation.

Relatively few natural product peptides have been described as RNA ligands. Although there might be many more out there, typical workflows for identifying the mode of action of a natural product do not consider RNA as a target but are strongly protein focused. The current rising interest in RNA targeting might alleviate that problem and it is likely that several other natural product cyclic peptides will be discovered. The examples described above do give an interesting insight into potent RNA binding. The ribosome targeting peptides surprisingly lack the presence of multiple positively charged amino acids and instead, achieve selective binding *via* stacking interactions as well as hydrogen bonding interactions of modified amino acids. These lessons can possibly be extrapolated to the optimization of peptide ligands or the design of new synthetic libraries for screening by including similar functional groups and heterocycles. Furthermore, one can use a naturally occurring cyclic peptide with intrinsic RNA binding affinity as a core scaffold for modification and testing against other RNA targets of interest. Such a screening could be performed by using methods similar to the Inforna platform described by the Disney group. Here, one first analyzes what type of RNA secondary structures a compound potently interacts with and then mines databases for RNAs that contain such structures (Velagapudi et al., 2016; Costales et al., 2020).

Although peptides have the capacity to target RNA in a selective manner, it is rarely demonstrated beyond a handful of tested RNA targets. It will advance the field significantly if information on selectivity will be provided in more detail especially in relation to the RNA secondary structure. Such an analysis is theoretically possible *via* techniques used for the identification of RNA targets of RBPs or RNA-binding small molecules. The advanced methods for peptide synthesis allow the straightforward preparation of a peptide pull-down probe by introducing immobilization handles. It can then be used to pull-down its RNA targets (after optional crosslinking), followed by reverse transcription and high-throughput sequencing to get an broad view of the selectivity of the ligand (Wang et al., 2018). Alternatively, a reactive module can be added to the peptide to promote crosslinking using the Chem-Clip approach that was effective in identifying miRNA targets of small molecules (Costales et al., 2019; Velagapudi et al., 2019). Besides addressing the selectivity on the RNA side, another important step in evaluating the RNA binding properties of a peptide is the peptide sequence itself. Scrambled versions of cyclic peptides where the amino acid order is randomized are useful to address whether the interaction is driven by the sequence or the general properties of the peptide. Such an approach is especially useful for the evaluation of polycationic peptides since their binding affinity might be driven by non-specific electrostatic interactions and is often lacking from the reports.

A critical aspect in successful ligand identification is the selection of the target itself (Warner et al., 2018; Costales et al., 2020). The flexible nature of RNA makes it hard to achieve potent binding as an entropic penalty will be involved upon binding if the flexibility of the RNA is reduced (Kwok et al., 2015; Bevilacqua et al., 2016). A peptide with a very high conformational stability might be able to overcome this issue especially if the peptide is able to generate a high number of specific interactions which significantly increases the enthalpic contribution to binding. In the absence of a stable tertiary structure, it is advantageous for a compound to interact with various RNA secondary structures simultaneously to achieve high potency. Since it is very difficult for monovalent small molecules to achieve this, cyclic peptides are an attractive alternative as RNA secondary structure binding ligands as they can span larger distances. That this can be achieved is illustrated by the high potency of the HIV TAR and RRE binding peptides which has not been reached by small molecules until now. A similar argument can be made for peptides **27**–**29** that target therapeutically relevant non-coding RNAs for which no small molecules have been reported.

The RNA-binding cyclic peptide described above demonstrate that the targeting of RNA is feasible and can be effective in cellular systems. However, no animal experiments or even clinical trials have been reported for any of the described molecules with the exception of some of the ribosome targeting antibiotic peptides. In various cases there seems to be a disconnect between affinity and cellular activity which is clearly demonstrated by the low cellular activity of peptide **8** (*K*_D_ < 0.18 nM). Such highly positively charged peptides could be ineffective due to issues with membrane permeability or they could get trapped in the endosomes after inducing endosomal uptake (LeCher et al., 2017). Another issue with this class could be strong non-specific interactions with other negatively charged biomolecules (i.e., ribosomal RNA, genomic DNA) lowering the effective free intracellular concentration. Cyclic peptides with a much more limited charge could therefore be more attractive compounds to test in animal models. The absence of such assays to date highlights the obstacles still to overcome using cyclic peptides as RNA ligands.

Although many lessons are still to be learned, the examples described above indicate that potent RNA binding can be achieved with macrocyclic peptides. We believe that by applying the right strategy to the right target it is highly conceivable that this class of molecules will be able to achieve the potency and selectivity required for therapeutic applicability. With the ongoing rise in interest in exploring RNA as a therapeutic target it will be important to explore a broad chemical space. The unique properties of cyclic peptides can make them a very suitable part of this space to explore further, and it is likely many more examples will appear in the near future.
